# Telemedicine perception and interest among medical students at the University of Sharjah, United Arab Emirates, 2023

**DOI:** 10.1186/s12909-023-04859-0

**Published:** 2023-11-22

**Authors:** Abdulaziz H. Albahri, Shatha A. Alnaqbi, Shahad A. Alnaqbi, Sarra Shorbagi

**Affiliations:** 1 Primary Healthcare Services Sector, Dubai Academic Health Corporation, Dubai, United Arab Emirates; 2 Department of Family Medicine and Community Medicine and Behavioral Sciences, College of Medicine, University of Sharjah, Sharjah, United Arab Emirates

**Keywords:** Telemedicine, Medical education, Medical curriculum, Distance learning

## Abstract

**Background:**

Telemedicine is becoming an integral part of healthcare. Training medical students in telemedicine is encouraged by many medical organizations. However, in the United Arab Emirates in particular, most medical schools have not incorporated it into their curriculum. Therefore, this study aims to assess medical students’ perceptions and interest in telemedicine teaching at the University of Sharjah, UAE.

**Methods:**

A questionnaire-based survey was built based on the current literature and was distributed to all medical students at the University of Sharjah between February and March 2023. The questionnaire assessed the participants for their demographic data, access to and use of digital devices, exposure to and beliefs related to telemedicine, and their medical school experience with distance learning and telemedicine. The data were analyzed via simple statistics, and the Chi-square test was used to assess the associated factors affecting the participants’ interest in receiving telemedicine teaching.

**Results:**

The questionnaire had a 70.4% (547/777) response rate. The mean age (SD) of the participants was 20.7 years (1.57), and the majority were female (68.4%). Over 98% of the students reported having easy access to and being comfortable with using computers and the internet. Most students (90.5%) believed that the medical school curriculum should include teaching in telemedicine; however, 78.2% of these students stated that it should be included as an elective course. The participants’ interest in receiving teaching in telemedicine had a statistically significant association with the following factors: being female, being familiar with telemedicine, having read literature on telemedicine, having beliefs that telemedicine is an opportunity to improve current medical practice, that its use should be encouraged, that it has an important role to play in healthcare, that it does not pose greater threat to current medical practice, having a preference to continue distance learning at medical school and having an interest in incorporating telemedicine in their future careers.

**Conclusions:**

It is an ideal time to incorporate telemedicine into the medical curriculum at the University of Sharjah with most students expressing interest in it. However, further research is needed to assess its applicability to other medical schools in the country and elsewhere.

**Supplementary Information:**

The online version contains supplementary material available at 10.1186/s12909-023-04859-0.

## Background

The twenty-first century has seen major advances in information and communication technologies (ICTs). This in turn has touched all aspects of human life, including healthcare [1]. One such important development in the field is telemedicine. While telemedicine is not a new concept in the history of medicine, the recent advancement in technology has enabled and facilitated its use at a broader range [2]. It is defined in simple terms as the distant delivery of patient care via various modes including audio, visual, text, or combinations of the aforementioned [3]. With its use, many challenges have been overcome, including the delivery of care to rural areas and places where certain medical expertise is needed. [4] On the other hand, it has created various additional challenges, such as potential threats to patient privacy and confidentiality [5].

Globally, various healthcare institutes have adopted the concept of telemedicine at various paces. However, since the coronavirus disease 2019 (COVID-19) pandemic, many countries and institutions have been forced to adopt telemedicine at a faster rate to deal with the surge of demand on healthcare, to monitor highly contagious ill patients, to follow up chronically ill patients at risk of contracting COVID-19, and for various other reasons [6, 7]. This in turn has created demand for enhancing healthcare professionals’ ability to utilize telemedicine efficiently in this critical period of humanity. The availability of resources, availability of technology, current healthcare infrastructure, and current telemedicine use have all affected how well countries have adapted to it [7].

On another note, universities and medical schools in particular, have been challenged by the pandemic and many of them have embraced the concept of distance learning [8]. With its similarities to telemedicine in terms of the remote mode of delivery and the need for ICT infrastructure with the staff that is trained and equipped well to utilize it, this has created a better opportunity to introduce the teaching and training of telemedicine to students at an early stage of their training [9]. As the demand on telemedicine has increased globally, the major focus has been to train the qualified healthcare professionals to utilize it rather than focusing on the next generation of doctors during their medical school training to be well prepared for their future careers. Major organizations in the US and Europe have encouraged the training of medical students and residents in telemedicine, a limited number of medical schools have embraced the training prior to the pandemic [10, 11]. Apart from Western countries, many medical schools in the developing and underdeveloped world have lagged behind in terms of introducing telemedicine teaching and training into their curriculum, and less is known about the interest of their students in terms of receiving training in this field [11, 12]. Moreover, there is limited research on the understanding of telemedicine and the factors associated with it among medical students in this part of the world. For curriculum developers and healthcare policy makers this aspect becomes crucial as they plan for training their future healthcare providers. In the United Arab Emirates (UAE) where the current study took place, telemedicine has been widely used during the pandemic with good ICT infrastructure in the country and accessibility to its residents [13–15]. However, the majority of medical schools in the country have not embraced the teaching of telemedicine officially in their curriculum. Once more, with very scarce data regarding the students’ interest in the field and the factors affecting them. The current study focused on students at the Emirate of Sharjah as will be further detailed in the methodology section. Accordingly, this study aimed to assess the perception of telemedicine among medical students at the Emirate of Sharjah, to explore the associated factors affecting their interest in receiving further teaching in the field, and the perceived barriers to receiving such teaching and training.

## Methodology

### Study setting and population

This study was conducted at the University of Sharjah Medical School. It is the only medical school in the Emirate of Sharjah and one of the major medical schools in the UAE. It runs a five-year program in which the first three years are preclinical and the latter two are clinical years. At the time of the study, the medical school did not have an official telemedicine training course incorporated into its curriculum. The study was conducted between February and March 2023. Paper copies of the questionnaire were distributed to all the students.

The sample size was calculated using the CDC Epi Info software v7.2 (available at the CDC website: www.cdc.gov/epiinfo). For a 95% confidence,3% margin of error and 50% expected frequency, the sample size was estimated to be 450 responses based on a total number of students at the school being 777.

### The study questionnaire

**Fig. 1:**
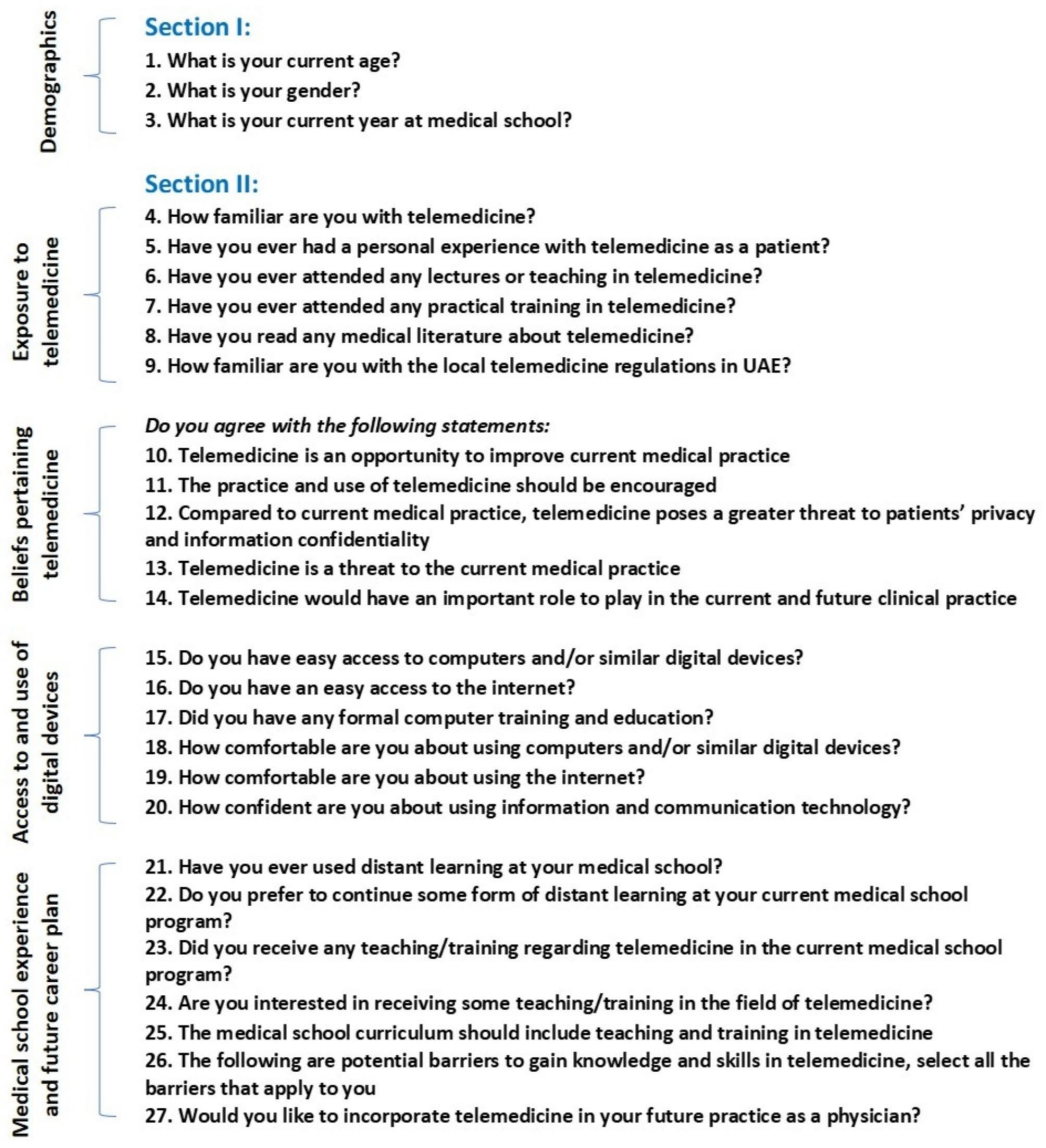
Study questionnaire and its associated domains

The questionnaire was designed based on a review of the available literature and published questionnaires [16–23]. There was a total of 27 questions divided into five major domains. The first domain asked the students for their demographics, including age, gender, and year in medical school. The second domain explored the students’ exposure to telemedicine by asking them about their familiarity with the field, their personal experience as patients, having had lectures or training in telemedicine, having read medical literature about the field, and their familiarity with the local regulations pertaining to it. The third domain explored the participants’ beliefs regarding whether they see telemedicine as an opportunity to improve current medical practice, that its practice should be encouraged, or that it is a threat to current medical practice and poses a greater threat to patients’ confidentiality and privacy, and whether they see it as having an important role in current and future medical practice. The fourth domain explored the background technical skills of the participants and their access to digital devices. This included questions related to access to computers and digital devices, access to the internet, having had computer training in the past, feeling comfortable using digital devices and the internet, and feeling confident using information and communication technology. The last domain explored the students’ medical school experience and their future career plan. This involved questions about having had distance learning in the past, whether they preferred to continue with some distance learning, whether they had any telemedicine teaching or training in their current program, their interest in receiving telemedicine teaching and training, whether telemedicine teaching and training should be incorporated into the medical school curriculum, barriers to gaining knowledge and skills in telemedicine, and whether they would like to incorporate telemedicine in their future practice as physicians. The domains are summarized in Fig. 1, and a sample of the questionnaire is included in the Additional files. The content and face validity of the questionnaire were examined by experts in the field. The questionnaire was piloted on 20 students and further modified to ensure clarity and relevance to the participants. The final analysis did not include the results of the pilot study.

### Data analysis

Statistical analysis was carried out using GraphPad Prism v9.5.1. In general, percentages and counts were used to explore the responses to the individual questions. Pearson’s chi-square test was used to explore the association between the participants’ interest in receiving teaching in telemedicine and the relevant associated factors, with a *p* value < 0.05 considered statistically significant.

### Ethical approval

The study was reviewed and approved by the Research Ethics Committee at the University of Sharjah (reference number REC-22-03-09). Participation in the study was voluntary, and all information was kept anonymous and confidential.

## Results

### Demographics and participants’ characteristics

There was a total of 547 participants with a response rate of 70.4% (547/777). More than two-thirds were female (374, 68.4%). The mean age was 20.7 (SD 1.57; range 18–26). Most of the participants were from the preclinical years 1–3 (382, 69.8%). The majority of the participants (more than 98%) stated having easy access to computers and the internet and feeling comfortable using them, as well as feeling confident using information and communication technology. Approximately 80% (436/547) of the students stated having formal computer training in the past.

While 97.1% (531/547) of the participants stated having some form of distance learning at medical school, only 52.3% stated preferring to continue with it at the current medical school program. Moreover, only 15.2% (83/547) reported receiving some form of teaching in telemedicine in their current medical school program. Most students stated that the medical school curriculum should include teaching and training in telemedicine (90.5%); however, only 21.8% preferred that it be included as a compulsory component of the program compared to 78.2% who preferred it be included as an elective course. The data on the participants’ characteristics are summarized in Table 1.

**Table 1:** Demographics and background characteristics of the study participants (n = 547)

Question	Answers	Number	%
***Demographics***			
**Age (in years)**	Mean (SD)	20.7 (1.57)	NA
	[minimum, maximum]	[18, 26]	
**Gender**	Male	173	31.6
	Female	374	68.4
**Year at medical school**	First year	140	25.6
	s year	116	21.2
	Third year	126	23.0
	Fourth year	81	14.8
	Fifth year	84	15.4
***Access to and use of digital devices***:			
**Have easy access to computers and/or similar digital devices**	Yes	539	98.5
	No	8	1.5
**Have an easy access to the internet**	Yes	541	98.9
	No	6	1.1
**Had any formal computer training and education**	Yes	436	79.7
	No	111	20.3
**Comfortable using computers and/or similar digital devices**	Comfortable (very comfortable- somewhat comfortable)	546	99.8
	Not comfortable	1	0.2
**Comfortable using the internet**	Comfortable (very comfortable- somewhat comfortable)	547	100
	Not comfortable	0	0
**Confident about using information and communication technology**	Confident (very confident- somewhat confident)	545	99.6
	Not confident	2	0.4
***Medical school experience***:			
**Had distance learning at medical school**	Yes	531	97.1
	No	16	2.9
**Prefers to continue some form of distance learning at the current medical school program**	Yes	286	52.3
	No	261	47.7
**Received teaching/training regarding telemedicine in the current medical school program**	Yes	83	15.2
	No	464	84.8
**Medical school curriculum should include teaching and training in telemedicine**	Yes	495	90.5
	*Yes, and it should be included as a compulsory component of the medical school program*	108	21.8
	*Yes, and it should be included as an elective course*	387	78.2
	No	52	9.5

### Interest in receiving telemedicine teaching and its associated factors

A total of 389 (71.1%) of the participating students expressed interest in receiving teaching in telemedicine. This was statistically significantly associated with gender (*X*^2^ [1] = 18.20, *p* < 0.0001), with a higher proportion of females than males interested in receiving telemedicine teaching. Age and year in medical school, on the other hand, did not show a significant association.

Regarding the exposure to telemedicine domain, 372 (68%) students expressed familiarity with telemedicine, and this had a significant statistical association with interest in receiving telemedicine teaching (*X*^2^ [1] = 12.46, *p* < 0.001). Additionally, only 70 (12.8%) participants stated having read literature about telemedicine; this also had a statistically significant association with interest in telemedicine teaching. On the other hand, having experienced telemedicine as a patient, having attended telemedicine teaching or practical training in the past, and being familiar with local telemedicine regulations had no statistical associations. Further details are described in Table 2.

**Table 2:** Factors associated with study participants’ interest in receiving telemedicine teaching (n = 547)

				Interested in receiving teaching in telemedicine						
				**Yes** [#,%]		**No** [#,%]				
				389 (71.1%)		158 (28.9%)		***Chi-Square test***		
**Question**	**Answers**	**Total**	**%**	**No.**	**%**	**No.**	**%**	**[df]**	***X*** ^**2**^ **value**	***P*** **value**
***Demographics***										
**Age**	18–19	150	27.4	104	69.3	46	30.7	2	0.56	0.76
	20–21	231	42.2	168	72.7	63	27.3			
	22 and older	166	30.3	117	70.5	49	29.5			
**Gender**	Male	173	31.6	102	59.0	71	41.0	1	18.20	< 0.0001
	Female	374	68.4	287	76.7	87	23.3			
**Year at medical school**	Preclinical (years 1–3)	382	69.8	269	70.4	113	29.6	1	0.30	0.58
	Clinical (years 4–5)	165	30.2	120	72.7	45	27.3			
***Exposure to telemedicine***										
**Familiarity with telemedicine**	Familiar (very familiar- somewhat familiar)	372	68.0	282	75.8	90	24.2	1	12.46	< 0.001
	Not familiar	175	32.0	107	61.1	68	38.9			
**Experience with telemedicine as a patient**	Yes	91	16.6	69	75.8	22	24.2	1	1.18	0.28
	No	456	83.4	320	70.2	136	29.8			
**Attended teaching in telemedicine**	Yes	77	14.1	60	77.9	17	22.1	1	2.02	0.16
	No	470	85.9	329	70.0	141	30.0			
**Attended practical training in telemedicine**	Yes	58	10.6	47	81.0	11	19.0	1	3.11	0.08
	No	489	89.4	342	69.9	147	30.1			
**Read any literature about telemedicine**	Yes	70	12.8	59	84.3	11	15.7	1	6.78	< 0.01
	No	477	87.2	330	69.2	147	30.8			
**Familiar with local telemedicine regulations**	Familiar (very familiar- somewhat familiar)	152	27.8	111	73.0	41	27.0	1	0.37	0.54
	Not familiar	395	72.2	278	70.4	117	29.6			
***Beliefs pertaining telemedicine***										
**Telemedicine is an opportunity to improve current medical practice**	Agree (strongly agree- somewhat agree)	506	92.5	377	74.5	129	25.5	1	37.79	< 0.0001
	Disagree (disagree- strongly disagree)	41	7.5	12	29.3	29	70.7			
**The practice and use of telemedicine should be encouraged**	Agree (strongly agree- somewhat agree)	493	90.1	371	75.3	122	24.7	1	41.64	< 0.0001
	Disagree (disagree- strongly disagree)	54	9.9	18	33.3	36	66.7			
**Telemedicine threatens patients’ privacy and information confidentiality**	Agree (strongly agree- somewhat agree)	336	61.4	229	68.2	107	31.8	1	3.72	0.05
	Disagree (disagree- strongly disagree)	211	38.6	160	75.8	51	24.2			
**Telemedicine is a threat to the current medical practice**	Agree (strongly agree- somewhat agree)	222	40.6	140	63.1	82	36.9	1	11.79	< 0.001
	Disagree (disagree- strongly disagree)	325	59.4	249	76.6	76	23.4			
**Telemedicine would have an important role to play in the current and future clinical practice**	Agree (strongly agree- somewhat agree)	496	90.7	376	75.8	120	24.2	1	57.00	< 0.0001
	Disagree (disagree- strongly disagree)	51	9.3	13	25.5	38	74.5			
***Medical school experience and future career plan***										
**Prefers to continue some form of distance learning at the current medical school program**	Yes	286	52.3	224	78.3	62	21.7	1	15.15	< 0.0001
	No	261	47.7	165	63.2	96	36.8			
**Received teaching/training regarding telemedicine in the current medical school program**	Yes	83	15.2	61	73.5	22	26.5	1	0.27	0.60
	No	464	84.8	328	70.7	136	29.3			
**Interested in incorporating telemedicine in future practice as a physician**	Yes	271	49.5	243	89.7	28	10.3	2	117.11	< 0.00001
	No	61	11.2	16	26.2	45	73.8			
	Undecided	215	39.3	130	60.5	85	39.5			

Regarding the participants’ telemedicine beliefs, over 90% of the participants believed that telemedicine is an opportunity to improve current medical practice, should be encouraged, and that it has an important role to play now and in the future. Only 222 students (40.6%) viewed it as a threat to current medical practice, and 336 (61.4%) viewed it as a threat to patients’ privacy and confidentiality. All of these beliefs had statistically significant associations with the interest in receiving telemedicine teaching apart from the belief that it is a threat to patients’ privacy, which was not significantly associated. Table 2 describes the details of the associations.

Over half of the students (52.3%) preferred to continue some form of distance learning in their current medical school program, and this preference was significantly associated with interest in receiving telemedicine teaching (*X*^2^ [1] = 15.15, *p* < 0.0001). On the other hand, having received some form of teaching in telemedicine in the current medical program did not show a statistically significant association. Finally, nearly half of the students (49.5%) expressed interest in using telemedicine in their future careers, while 39.3% were undecided and 11.2% were not interested. This is associated with the interest in receiving telemedicine teaching (*X*^2^ [2] = 117.11, *p* < 0.00001), with a higher proportion of students interested in using telemedicine in their future career also being interested in receiving telemedicine teaching. Further details of the associations are summarized in Table 2.

### Barriers to gaining knowledge and skills in telemedicine

As summarized in Table 3, a lack of appropriate educational programs is perceived as the top barrier (329, 60.1%), followed by a lack of qualified staff (313, 57.2%). Lack of access to the technology and financial constraints were perceived as barriers by 19.7% and 19.0% of the participants, respectively. Some participants described other barriers (data not shown in table), such as lack of time in a busy medical school curriculum and telemedicine being a new unfamiliar field.

**Table 3:** Perceived barriers to gaining knowledge and skills in telemedicine (n = 547)

Barriers	Number	%
Lack of appropriate educational programs	329	60.1
Financial constraints	104	19.0
Lack of sufficient access to technology	108	19.7
Lack of qualified and experienced instructors in telemedicine	313	57.2
Others	23	4.2

*Participants can select more than one option; thus, percentages do not add up*

## Discussion

In the current study, the majority of students expressed interest in receiving teaching in telemedicine, with over 71% interested. This highlights an opportunity for curriculum developers as more students realize and appreciate the importance of telemedicine and its value in the healthcare field, especially after the COVID-19 pandemic [24]. Of note, the vast majority of students stated having access to and being comfortable with using digital technology. The UAE is known to have a good ICT infrastructure supporting most of the industries and fields in the country, including healthcare [14]. Whether similar views and interests are maintained in countries where there is less access to and familiarity with modern technology remains to be elucidated by relevant studies in the targeted population. One study among medical students in Nepal by Kunwar et al. showed that students shared similar views and interests in telemedicine to a certain degree as our study, and another study in Saudi Arabia highlighted the same [25, 26]. Nonetheless, further studies are still needed in more countries and regions globally [27].

Furthermore, over 90% of the students stated that the medical school curriculum should include teaching and training in telemedicine. However, most of these students (78.2%) stated that it should be incorporated as an elective course. This hesitation to have it as a compulsory course highlights the reality of busy curricula in most medical schools. When asked about the barriers to gaining knowledge and skills in telemedicine, some of the written responses included statements such as time constraints and a busy curriculum. Further research would be needed to explore this area and elucidate the best method of delivery, whether to keep it as a compulsory or as an elective course, and to explore in more details the hesitation among students to have it as a compulsory one. Moreover, it would be of interest to see if similar views are shared by students in other parts of the country and elsewhere globally. Innovation in the teaching of telemedicine is needed, whether to incorporate it along with other subjects and clinical skills training or to keep it as a separate subject to be taught independently is another question worth further research and exploration [28]. Regardless of the best method of delivery, which was not the focus of the current study, evaluation of the program success and outcomes for current students and postgraduates would be needed to determine the success of any such programs [29].

Regarding the factors associated with students’ interest in telemedicine teaching, female students expressed greater interest in telemedicine. The reasons are open for speculation, as they were not explored in the current study, but generally, females seem to have greater positive attitude toward patient-doctor communication and patient-centered care, as shown in other studies [30]. Nevertheless, further studies are needed to elucidate the reasons behind this discrepancy and whether it applies to other cultures and other parts of the world or is a unique aspect of this region.

Students who described being familiar with telemedicine and having read literature about it, showed greater interest in receiving further teaching. This in itself might be explained by the fact that such students had prior interest in the subject and thus explored it further. At the same time, this might be a point for curriculum developers to explore and expose students to such literature to foster their interest in the subject prior to fully enrolling them in such courses, especially if the school decides to keep the course as an elective one [24, 28].

Most of the students had positive beliefs regarding telemedicine, with over 90% of them believing that it is an opportunity to improve current medical practice, that its use should be encouraged and that it will have an important role to play in the future. This high proportion of students with such positive beliefs might have been influenced by the COVID-19 pandemic and appreciating the application of telemedicine during the pandemic [27, 31]. Additionally, having technical skills and living in a country with strong technological infrastructure might have contributed to these beliefs as well. Whether such beliefs are maintained throughout the students’ training journey and whether practicing physicians hold similar beliefs need to be further explored in future studies [30].

### Study limitations

This study has some limitations. First, it suffers from the inherent limitations of cross-sectional questionnaire-based surveys, including recall bias and response bias, since the data are analyzed based on the reported answers of the participants. Second, this study is based on students’ participation from a single medical school; thus, the generalizability of the data to other medical schools in the country and elsewhere is limited until further studies are conducted. Last, this study samples the students’ views at a single point in time; thus, it does not assess whether their views change over time as they progress through medical school and gain further clinical experience; however, an attempt to analyze the difference between the clinical and preclinical years was conducted.

## Conclusions

With increasing demand for telemedicine locally and globally and with rapid advancement in medical technology, pressure is increasing on medical schools to adapt to such changes and prepare their students for their future careers. In countries with good technological infrastructure, such as the UAE, most of the population has the basic needed technical skills, which applies to medical students, as described in our study. Accordingly, this is an advantage that such medical schools can build on to develop a structured program that will advance these skills and refine them to the medical field. It is no surprise that the medical curriculum in any school is quite busy, but the willingness and interest of students to incorporate telemedicine highlights the importance of the continuous reexamination of the medical curriculum to adapt to the changing world, population demand, and medical technology. While our study addressed a limited population in a single university, it does pave the way for further studies in the field and in other universities locally and internationally. Furthermore, it provides guidance to curriculum developers in terms of areas to focus on and areas where they might face challenges and resistance from the students.

### Electronic supplementary material

Below is the link to the electronic supplementary material.

Supplementary Material 1: Telemedicine is the use of telecommunications technology (such as video call, telephone, e-mail, etc.) in the remotediagnosis and treatment of patients. The current questionnaire aims to assess your knowledge and perceptions regarding telemedicine.

## Data Availability

All data generated or analyzed during this study are included in this published article. Any additional datasets used and/or analyzed during the current study and not presented in the current paper are available from the corresponding author on reasonable request.

## List of abbreviations

COVID-19: coronavirus disease 2019
ICT: Information and communication technology
UAE: United Arab Emirates
