# Supplementary material for: Telemedicine perception and interest among medical students at the University of Sharjah, United Arab Emirates, 2023
Source: BMC Med Educ. 2023 Nov 22;23:892. doi: 10.1186/s12909-023-04859-0 (PMC10666329; doi:10.1186/s12909-023-04859-0)
Supplement: Supplementary file 1 — Supplementary Material 1: Telemedicine is the use of telecommunications technology (such as video call, telephone, e-mail, etc.) in the remotediagnosis and treatment of patients. The current questionnaire aims to assess your knowledge and perceptions regarding telemedicine. [file 12909_2023_4859_MOESM1_ESM.pdf]

Telemedicine Perception and Interest among Medical Students at the University of Sharjah, UAE, 2023

Telemedicine is the use of telecommunications technology (such as video call, telephone, e-mail, etc.) in the remote diagnosis and treatment of patients. The current questionnaire aims to assess your knowledge and perceptions regarding telemedicine.

**Section I: Demographics**	
**1. What is your current age?**	[……….] (*write your age in numbers)*
**2. What is your gender?**	☐ Male ☐ Female
**3. What is your current year at medical school?** [*do not count foundation year if done*]	☐ First year ☐ Second year ☐ Third year ☐ Fourth year ☐ Fifth year
**Section II: Questions**	
**4. How familiar are you with telemedicine?**	☐ Very familiar ☐ Somewhat familiar ☐ Not familiar
**5. Have you ever had a personal experience with telemedicine as a patient?**	☐ Yes ☐ No
**6. Have you ever attended any lectures or teaching in telemedicine?**	☐ Yes ☐ No
**7. Have you ever attended any practical training in telemedicine?**	☐ Yes ☐ No
**8. Have you read any medical literature about telemedicine?**	☐ Yes ☐ No
**9. How familiar are you with the local telemedicine regulations in UAE?**	☐ Very familiar ☐ Somewhat familiar ☐ Not familiar
Do you agree with the following statements:	
**10. Telemedicine is an opportunity to improve current medical practice**	
☐ Strongly agree ☐ Agree ☐ Somewhat agree ☐ Disagree ☐ Strongly disagree	
**11. The practice and use of telemedicine should be encouraged**	
☐ Strongly agree ☐ Agree ☐ Somewhat agree ☐ Disagree ☐ Strongly disagree	
**12. Compared to current medical practice, telemedicine poses a greater threat to patients' privacy and information confidentiality**	
☐ Strongly agree ☐ Agree ☐ Somewhat agree ☐ Disagree ☐ Strongly disagree	
**13. Telemedicine is a threat to the current medical practice**	
☐ Strongly agree ☐ Agree ☐ Somewhat agree ☐ Disagree ☐ Strongly disagree	
**14. Telemedicine would have an important role to play in the current and future clinical practice**	
☐ Strongly agree ☐ Agree ☐ Somewhat agree ☐ Disagree ☐ Strongly disagree	
Access to and use of digital devices:	

Continue in next page…

15. Do you have an easy access to computers and/or similar digital devices?

☐ Yes ☐ No

16. Do you have an easy access to the internet?

☐ Yes ☐ No

17. Did you have any formal computer training and education?

☐ Yes ☐ No

18. How comfortable are you about using computers and/or similar digital devices?

☐ Very comfortable ☐ Somewhat comfortable ☐ Not comfortable

19. How comfortable are you about using the internet?

☐ Very comfortable ☐ Somewhat comfortable ☐ Not comfortable

20. How confident are you about using information and communication technology?

☐ Very confident ☐ Somewhat confident ☐ Not confident

Medical school experience:

21. Have you ever used distance learning at your medical school?

☐ Yes ☐ No

22. Do you prefer to continue some form of distance learning at your current medical school program?

☐ Yes ☐ No

23. Did you receive any teaching/training regarding telemedicine in the current medical school program?

☐ Yes ☐ No

24. Are you interested in receiving some teaching/training in the field of telemedicine?

☐ Yes ☐ No

25. The medical school curriculum should include teaching and training in telemedicine

☐ Yes, and it should be included as a **compulsory** component of the medical school program

☐ Yes, and it should be included as an **elective** course

☐ No

26. The following are potential barriers to gain knowledge and skills in telemedicine, select all the barriers that apply to you:

☐ Lack of appropriate educational programs

☐ Financial constraints

☐ Lack of sufficient access to technology

☐ Lack of qualified and experienced instructors in telemedicine

☐ Others (specify):

27. Would you like to incorporate telemedicine in your future practice as a physician?

☐ Yes ☐ No ☐ Undecided

Thank you for your participation!
